# Detecting gaze shifts of moving observers in dynamic environments

**DOI:** 10.3758/s13428-026-02972-8

**Published:** 2026-04-21

**Authors:** Ashkan Nejad, Andrea Ghiani, Frans W. Cornelissen, Eli Brenner

**Affiliations:** 1 Department of Research and Improvement of Care, Royal Dutch Visio, Huizen, The Netherlands; 2 Laboratory of Experimental Ophthalmology, University Medical Center Groningen, Groningen, The Netherlands; 3 Department of General Psychology, University of Padova, Padova, Italy; 4 Department of Movement Sciences, Vrije Universiteit Amsterdam, Amsterdam, The Netherlands

**Keywords:** Mobile eye-tracking, Gaze shift detection, Saccades, Dynamic environments, Machine learning

## Abstract

Eye-tracking using head-mounted systems in natural, dynamic settings presents unique challenges for accurately detecting gaze shifts (saccades). Unlike controlled, screen-based scenarios, mobile eye-tracking involves constant shifts in visual scenes with respect to the head due to head and body movements, complicating gaze event classification. This paper systematically evaluates several threshold- and machine-learning-based gaze-shift detection algorithms on a manually labeled dataset collected from participants walking freely outdoors. Our findings indicate that conventional threshold-based methods, despite their sensitivity to parameter settings, outperform contemporary pre-trained machine learning methods when applied to real-world dynamic conditions without retraining. However, we also demonstrated that although machine-learning-based methods perform poorly on unseen dynamic data, their performance improves substantially when they are retrained on data that closely matches the testing conditions. Moreover, we introduce a novel probabilistic approach, the Ranking method, that integrates both eye movement and visual scene information, achieving performance comparable to inter-annotator agreement, outperforming previous methods. We also report observing some gaze behaviors during manual annotation that do not fit within the classical gaze event categories, highlighting why classifying gaze events in unconstrained natural scenarios is more complex than doing so for screen-based tasks. Our work provides insights suggesting how one could improve gaze event classification performance in real-world environments in the future.

## Introduction

Eye movements provide a window into human visual processing, attention, and cognition. In everyday tasks such as walking, driving, or making tea and sandwiches (Brenner et al., 2024; Land, 1992; Land & Hayhoe, 2001), our momentary gaze direction reveals what information we prioritize. Recent advances in head-mounted eye trackers enable researchers to monitor gaze behavior in dynamic, natural settings outside the lab. Unlike traditional screen-based setups, which assume a static head and a fixed display, head-mounted systems track gaze while allowing free head and body movements. This means the visual scene continuously shifts with respect to the head, complicating the analysis of eye-tracking data. Studying gaze in such unconstrained settings is crucial for understanding vision in a more natural context, but it also poses new challenges for interpreting eye movement signals.

An essential step in analyzing raw gaze data is identifying gaze events, such as fixations and gaze shifts (saccades). Fixations occur when gaze is directed at an item, stabilizing it on the fovea for detailed inspection. In contrast, gaze shifts are rapid movements that reposition gaze from one item to another to gather information about multiple items in the environment (Kothari et al., 2020). In this paper, gaze shifts (or saccades) are defined broadly to include both voluntary rapid eye movements and fast phases of the vestibulo-ocular reflex (VOR).

Identifying gaze shifts and fixations is an essential component of the analysis for various applications, such as diagnosis (Cesari et al., 2022; Stuart, Galna, Lord, Rochester, & Godfrey, 2014), human-computer interaction (Ohno, Mukawa, & Yoshikawa, 2002), understanding drivers’ behaviors (Sharma & Chakraborty, 2024), and rehabilitation (Gestefeld, Koopman, Vrijling, Cornelissen, & de Haan, 2020). In all these applications, reliable event identification ensures that subsequent interpretations of gaze data are based on solid and meaningful measures (Hooge, Niehorster, Nyström, Andersson, & Hessels, 2022).

### Previous methods

#### Threshold-based methods

Various methods have been developed to detect and classify gaze or eye-movement events automatically. Historically, the most widely used approaches have been threshold-based algorithms, which apply decision rules to captured data based on a predetermined threshold value. For example, the velocity-threshold identification (I-VT) algorithm classifies points as saccades if the eye motion velocity exceeds a certain threshold (Salvucci & Goldberg, 2000). Conversely, the dispersion-threshold identification (I-DT) algorithm looks at the spatial dispersion of gaze points (Salvucci & Goldberg, 2000). If consecutive samples stay within a small area for some minimum duration, they are labeled a fixation. Any sample outside that area is classified as part of a saccade. Such methods are appealing for their simplicity, but threshold-based detectors come with notable limitations. They require careful tuning of the threshold, which may not generalize across individuals or recording conditions (Birawo & Kasprowski, 2022). If the threshold is set too low or too high, a saccade might be misclassified as a fixation or vice versa. To improve detection, researchers have suggested adding extra criteria. Some approaches combine both velocity and dispersion rules, while others introduce a second, lower velocity threshold to detect prolonged slow movements, such as when the eyes track a moving object (Komogortsev & Karpov, 2013).

Hooge and Camps (2013) introduced measures like scan path entropy and novel visualization techniques to better capture scanning behavior, highlighting the improved event detection performance. Threshold-based methods laid the groundwork for eye-movement event detection and continue to be used widely, but their simplicity may become a drawback in complex, noisy, or dynamic viewing scenarios.

#### Machine-learning-based methods

In recent years, machine-learning-based approaches have emerged to overcome the limitations of thresholds. Instead of using predefined rules, these methods learn to recognize patterns of eye-movement events from data. Some approaches first compute features, such as eye movement velocity or acceleration over a window and feed these into a classifier to train them to recognize the provided event types (Zemblys, Niehorster, Komogortsev, & Holmqvist, 2018). In contrast, deep learning has opened up new possibilities by enabling end-to-end event detection, eliminating the need to extract predefined features. GazeNet’s approach (Zemblys, Niehorster, & Holmqvist, 2019) applies a deep convolutional neural network directly to raw eye-tracking signals to classify each sample as being part of a saccade, fixation, or smooth pursuit. Another deep-learning-based approach is the Online Eye-Movement Classification (OEMC) method (Elmadjian, Gonzales, Costa, & Morimoto, 2023), which employs a Temporal Convolutional Network to enable event detection in real time. OEMC’s model predicts the eye movement event for a sample using only the information in the past samples, enabling online use, and demonstrating a higher performance than previous deep learning-based methods on screen-based recordings. However, the deep learning-based methods typically demand large amounts of labeled training data, which may not always be available.

The previously mentioned algorithms were all developed and tested using eye-movement signals from screen-based experiments, where the participants remained stationary with respect to the screen. While some researchers argue that existing methods should still be applicable to dynamic natural viewing conditions (Zemblys et al., 2019), others argue that environmental changes and body motion must be accounted for when detecting gaze events (Kothari et al., 2020; Nejad, de Haan, Heutink, & Cornelissen, 2024).

Kothari et al. (2020) designed a method to account for head motion when classifying gaze events that relies on IMU sensors and depth cameras to estimate how the head has moved and to evaluate the layout of the environment. However, such extra devices are not always available. A recent method called Automatic Classification of Gaze Events in Dynamic Natural Viewing (ACE-DNV), complemented eye-tracker signals by using the scene video to account for observer motion (Nejad et al., 2024). ACE-DNV achieved comparable performance to prior methods tested on the Gaze-in-Wild dataset (Kothari et al., 2020) while only using the data available from the mobile eye tracker. By using a random forest classifier, it was able to determine the importance of different hand-crafted features based on different tasks conducted by the participants. However, a key limitation of machine learning and deep learning-based methods is that their performance often may not generalize beyond the dataset on which they were trained. This is because the models may learn features that are specific to particular aspects of the dataset, such as lighting conditions, the nature of the task, or the specific eye-tracking device used.

### Aim and approach

To better understand how such methods perform outside controlled lab conditions and beyond the dataset on which they have been trained, there is a need for a more systematic evaluation of gaze event detection algorithms when applied to data from head-mounted eye trackers in dynamic, natural viewing environments. Previous studies have benchmarked eye-movement event detection algorithms extensively in traditional screen-based contexts (Andersson, Larsson, Holmqvist, Stridh, & Nyström, 2017; Birawo & Kasprowski, 2022; Startsev & Zemblys, 2023). However, to our knowledge, no study so far has assessed how these algorithms perform when the observer is moving around freely in the real world. Such an evaluation is warranted, as in mobile eye-tracking, the interplay of eye and head movements, the lower sampling rates of eye-trackers and scene cameras, and the lack of a fixed reference frame can all impact the event classification performance.

This paper aims to fill this gap by presenting a comparison of existing eye-movement and gaze event identification techniques on data from a natural dynamic viewing task, collected using a head-mounted eye tracker. In a previous study, we showed that visual information in the central part of vision could be as important as eye-movement information for classification of gaze events (Nejad et al., 2024). In addition to testing existing methods, we also introduce a new algorithm tailored for gaze shift detection in mobile settings that relies on the visual information near where gaze is directed as well as the eye movements themselves to classify gaze as saccades or fixations. We will refer to this new method, which integrates both eye movement dynamics and scene information, as the Ranking algorithm.

The main contributions of this study are as follows:

- We systematically compare a broad range of existing threshold-based and machine-learning-based gaze shift identification methods, alongside a newly proposed Ranking algorithm.
- We introduce a manually labeled dataset collected during outdoor walking, using head-mounted eye trackers, capturing gaze behavior under realistic, dynamic conditions.
- To assess generalizability, we additionally evaluate all methods on an independent, previously published dataset with greater variability in participant activities (Drews & Dierkes, 2024).
- By testing methods across multiple datasets, tasks, and annotators, we examine how reliably gaze shift identification techniques perform outside well-controlled laboratory settings.

## Datasets

In this section, we describe the datasets used to evaluate the gaze shift identification methods. We used two datasets: 1) a dataset that we ourselves collected and labeled, and 2) the dynamic recordings from the dataset of Drews and Dierkes (2024) that they collected and labeled. The next subsections describe our dataset in terms of participants, data acquisition procedures, and the manual labeling protocol. We then describe how the dataset of Drews and Dierkes (2024) was used in our study.

### Our dataset

#### Participants

The eye tracking data of six participants (mean age: 30 years old, age range: 26–32 years old, all male) of an already-existing dataset were used (Ghiani, Mann, & Brenner, 2024). The data that was used for the analysis is publicly available on Open Science Framework (DOI 10.17605/OSF.IO/45WBH). The experiment was conducted with the approval of the Scientific and Ethical Review Board of the Faculty of Behavioural and Movement Sciences at Vrije Universiteit Amsterdam (file VCWE-2021-035). All participants provided written informed consent. The participants included in this study provided separate informed consent to make their video data public.

#### Data collection

**Fig. 1:**
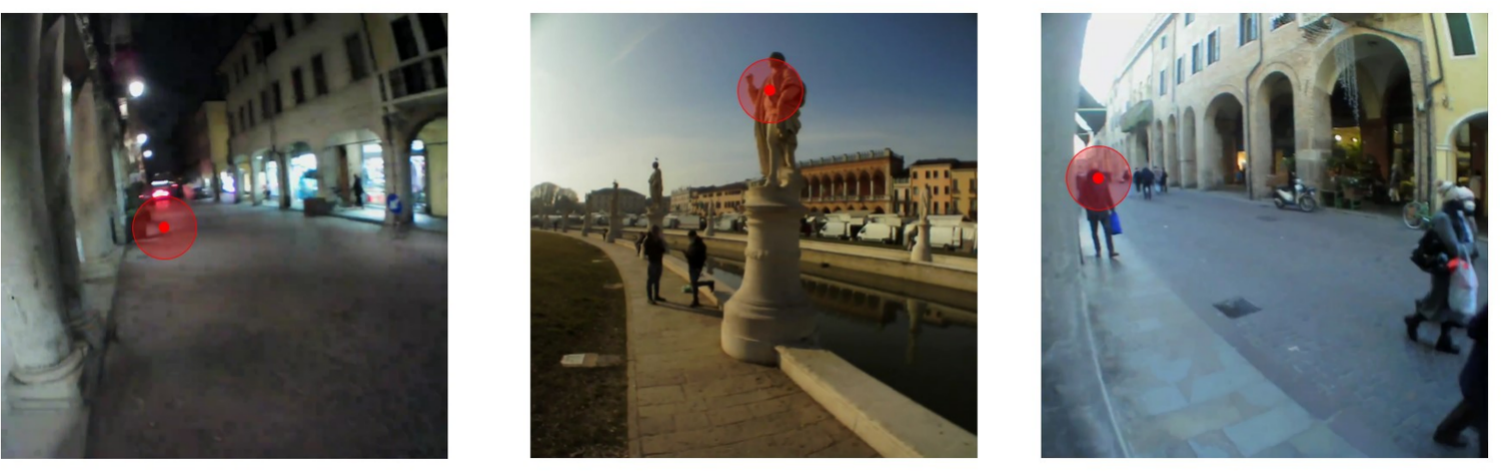
Three example frames from our dataset, each showing the corresponding gaze location, visualized with red circles

Pupil Invisible eye-tracking glasses (Pupil Labs, GmbH) were used to measure gaze direction at 200 Hz and record video footage of the scene from the participant’s viewpoint at about 30 Hz. This wearable eye tracker requires no calibration. Participants were instructed to walk to a square where they were to examine a group of statues for 2–3 min before returning. Walking to the square, looking at the statues, and then walking back, together took approximately 8 minutes, resulting in a total of about 45 min of eye-tracking recordings. This task naturally involved both head and body movements, providing dynamic motion data for our recordings. The dataset used in this study was originally collected by Ghiani et al. (2024). For the current study, we manually annotated a portion of this data. The gaze shift labels are publicly available together with the data itself on our online repository (Section “Availability of Data and Code”). Figure 1 shows three example frames from recordings in our dataset, each displaying the corresponding gaze location.

**Fig. 2:**
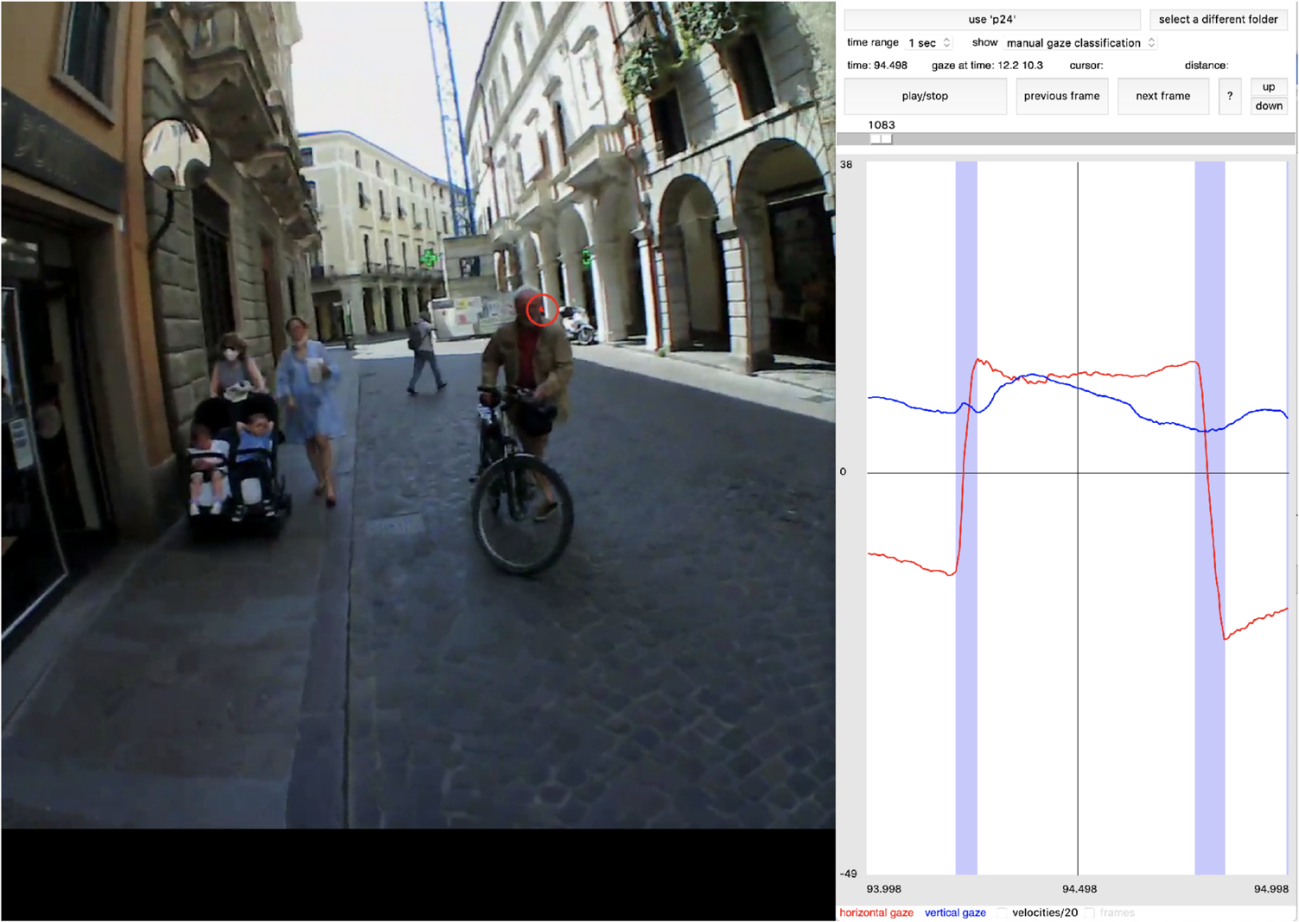
The interface used by the labelers to annotate sequences of gaze shifts in the recordings, while simultaneously viewing the eye movement traces and the corresponding scene video

#### Manual labeling

Manual annotation was performed by two coders (two of the authors, AG and EB), who independently labeled segments of eye movement traces as saccades. EB and AG did not confer or coordinate during the annotation process to ensure independent coding, but shared a common understanding of what constitutes a saccade based on their prior experience. Their only agreement was to identify and mark gaze shifts throughout the recordings.

The coders viewed the gaze trace centered on each image frame, alongside the corresponding video frame and the gaze position at that moment. They navigated through the recording frame by frame and marked saccade segments at the full resolution of the eye-tracking data (5 ms; 200 Hz). Coders were allowed to adjust their selections as needed throughout the annotation process. Figure 2 illustrates the interface used by our labelers for annotating the recordings. Our labelers did not identify segments as blinks; instead, they relied on Pupil Invisible’s blink detector.

### Drews and Dierkes dataset

In this study, we also used the dynamic activity recordings from the dataset by Drews and Dierkes (2024) to further ascertain the generalizability of our findings, ensuring that our evaluation of the algorithms, especially of the Ranking method, were not unduly biased by the fact that we ourselves were the manual annotators. For their dataset, Drews and Dierkes labeled fixations rather than saccades. However, since their distinction between saccades and fixations aligns with ours, most of what they code as gaps between fixations coincides with what we interpret as saccades (gaze shifts). The only exception is in the treatment of blinks. In the original study, Drews and Dierkes (2024) did not explicitly remove blinks, as these do not resemble fixations and thus would not be misclassified as such. Neither by the coder, nor by the algorithm used to detect fixations. However, blinks can be confused with gaze shifts. For our own dataset, we used Pupil Labs’ blink detection, which relies directly on eye images to identify blinks. However, this was not possible for the Drews and Dierkes dataset, as eye image videos were not provided. To address this, we assumed that any gaps between fixations that lasted longer than 100 ms must be blinks rather than gaze shifts. This value of 100 ms was chosen because saccades in classical main-sequence data are shorter than 100 ms unless they have an exceptionally large amplitude (Bahill, Clark, & Stark, 1975) and blinks typically last at least 150 ms (Stern, Walrath, & Goldstein, 1984). After recoding gaps as either saccades or blinks according to this simple criterion, the Drews and Dierkes dataset was processed using the same procedures as our own. In total, this dataset contributed approximately 12 min of eye-tracking data.

## Methods

In this section, we provide an overview of the gaze shift identification methods evaluated in our study. Each subsection briefly describes one of the selected algorithms, covering both traditional threshold-based approaches and modern machine-learning-based methods. We also introduce our new approach, which we will refer to as the Ranking method. This method was specifically designed for gaze shift detection in head-mounted eye tracking. Before detailing these methods, we describe the criteria used to select gaze-shift identification methods, our evaluation process, and the performance metrics.

### Performance evaluation

In our evaluation procedure, we compared the predicted labels to the reference labels at two levels of detail: per sample and per event. For the event-level analysis, we adopted the procedure proposed by Hoppe and Bulling (2016), which employs a majority-voting event matching strategy and uses the F1-score as the performance metric. The F1-score is calculated using the number of true-positive (TP), false-positive (FP), and false-negative (FN) predictions:

1 $$\begin{aligned} F1=\frac{TP}{TP+{(FP+FN)}/2}. \end{aligned}$$

To compute the sample-level F1-score, we compare each predicted sample in the continuous data stream to the corresponding ground truth label. Note that we consider the human classifier’s labels as the ground truth, although they are obviously quite subjective. For the event-level F1-score, each ground truth event is assigned a predicted label based on the most frequent class among the model’s predictions within that event, as explained below.

**Fig. 3:**
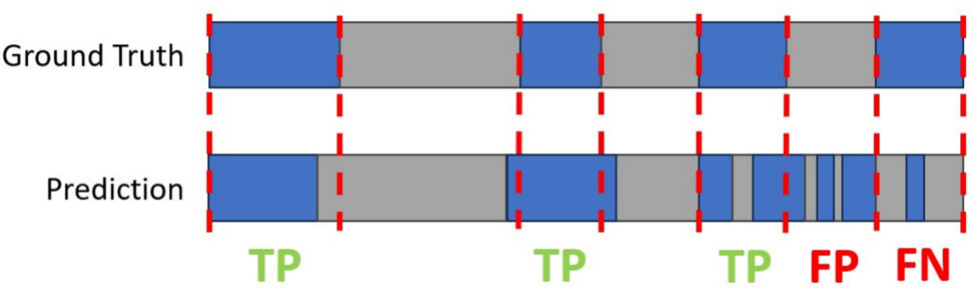
Illustration of true-positives, false-positives, and false-negatives in the majority-voting event matching approach. *Blue regions* indicate segments of samples labeled as saccades. *Red dotted lines* mark the boundaries of ground-truth saccade events. Note that slight discrepancies in the positions of the boundaries do not affect the categorization

The event-level approach treats each continuous labeled segment of data in its entirety. If the majority of the sample predictions in the corresponding region matched the ground truth label for a segment, the prediction was considered to be a correct identification. Conversely, any falsely identified or missed event contributed to the false-positive or false-negative count, respectively. We then calculated the overall F1-score on the basis of these counts. This event-level scoring method is generally more lenient than a sample-level approach, as it is less sensitive to brief discontinuities or inconsistencies in the predicted labels, which are common in manually annotated gaze data (Sharma & Chakraborty, 2025). Figure 3 provides a schematic visualization of TP, FN, and FP when comparing manual labels (ground truth) to predictions from a saccade detector.

By examining both sample-level and event-level performance, we obtained a comprehensive assessment of how accurately various measures capture short-term sample classifications as well as complete structured events in the data. We implemented our performance evaluation based on the code from the work by Elmadjian et al. (2023).

The known methods evaluated in this study are divided into two categories. The first category, threshold-based methods, requires initial parameter settings. The parameters are typically determined through a form of subjective validation. In our implementation, we optimized the thresholds using a representative subset for each dataset using one-sixth of the data: one recording for our own dataset, and four recordings for the Drews and Dierkes dataset. We then applied the selected values consistently across all recordings. The reported minimum fixation duration thresholds used with these algorithms depend strongly on the recording setup and task. In previous studies that involved mobile eye-tracking, this threshold was usually set within the range of 40 ms to 240 ms (Drews & Dierkes, 2024). Due to this wide range, we optimize this parameter for each condition separately.

The second category, machine-learning-based methods, was first evaluated using the authors’ publicly available pre-trained models. In the second phase, these models were retrained and tested using a k-fold cross-validation procedure to assess their performance when trained and tested on the same dataset. This two-step evaluation allows us to assess the generalizability of machine learning models to novel but similar data, a crucial property, given that ground-truth labels are often unavailable and manual annotation is time-consuming and labor-intensive. Retraining the models in this controlled setting provides insight into their potential when only limited annotated data is available for fine-tuning in a new study.

For this evaluation process, we used recording-wise six-fold cross-validation. We partitioned the set of recordings into six non-overlapping folds (groups) or recordings. In each iteration, we held out one fold (1/6 of the recordings) for testing and trained on the remaining five folds. Then, the results were averaged over the six test folds. The k-fold cross-validation was conducted independently for each dataset and for each annotator, with the results reported separately.

For additional validation, we tested the generalizability of the machine-learning-based methods across datasets and labelers. Specifically, we trained each method on the full dataset annotated by a single labeler, and then evaluated its performance across all three combinations of datasets and labelers. This approach allowed us to explore how well each model transfers across data sources and annotator perspectives. Such a transfer is necessary for most real-world use cases.

### Selecting gaze shift identification methods

The methods included in this study were chosen from common gaze or eye-movement event identification algorithms, based on five criteria: 1) First, we ensured that the evaluated method could be applied to head-mounted eye tracking in natural, dynamic settings. In mobile eye tracking, the observer’s visual environment is constantly changing, and no specific viewing distance can be assumed. Therefore, we selected algorithms that can be applied in free-viewing conditions without requiring fixed references. We thus excluded methods designed specifically for screen-based recordings, which often rely on predefined screen distances or screen size constraints. 2) The second important criterion in our selection process was the availability of publicly shared implementations. By choosing methods with existing, openly accessible code, we aimed to ensure the reproducibility of our results and facilitate further comparative research. 3) Additionally, the selected algorithms either explicitly classify saccades among other gaze or eye-movement events or distinguish between fast and slow phases of eye movement, where the fast phase corresponds to saccades or gaze shifts. 4) We also excluded methods that rely on IMU sensors or depth cameras. 5) Finally, we excluded methods that have previously been shown to have inferior performance compared to other available approaches in our candidate list that are based on similar principles. This process resulted in a final selection of six saccade and gaze shift identification methods, comprising three threshold-based approaches and three machine-learning-based approaches. The threshold-based approaches (Section “Threshold-based methods”) include Identification by Dispersion Threshold (I-DT), Identification by Velocity Threshold (I-VT) and the Moving Window methods. The machine-learning-based approaches (Section “Machine-learning-based methods”) include Online Eye-Movement Classification with temporal convolutional networks (OEMC), GazeNet, and Automatic Classification of gaze Events in Dynamic Natural Viewing (ACE-DNV).

**Fig. 4:**
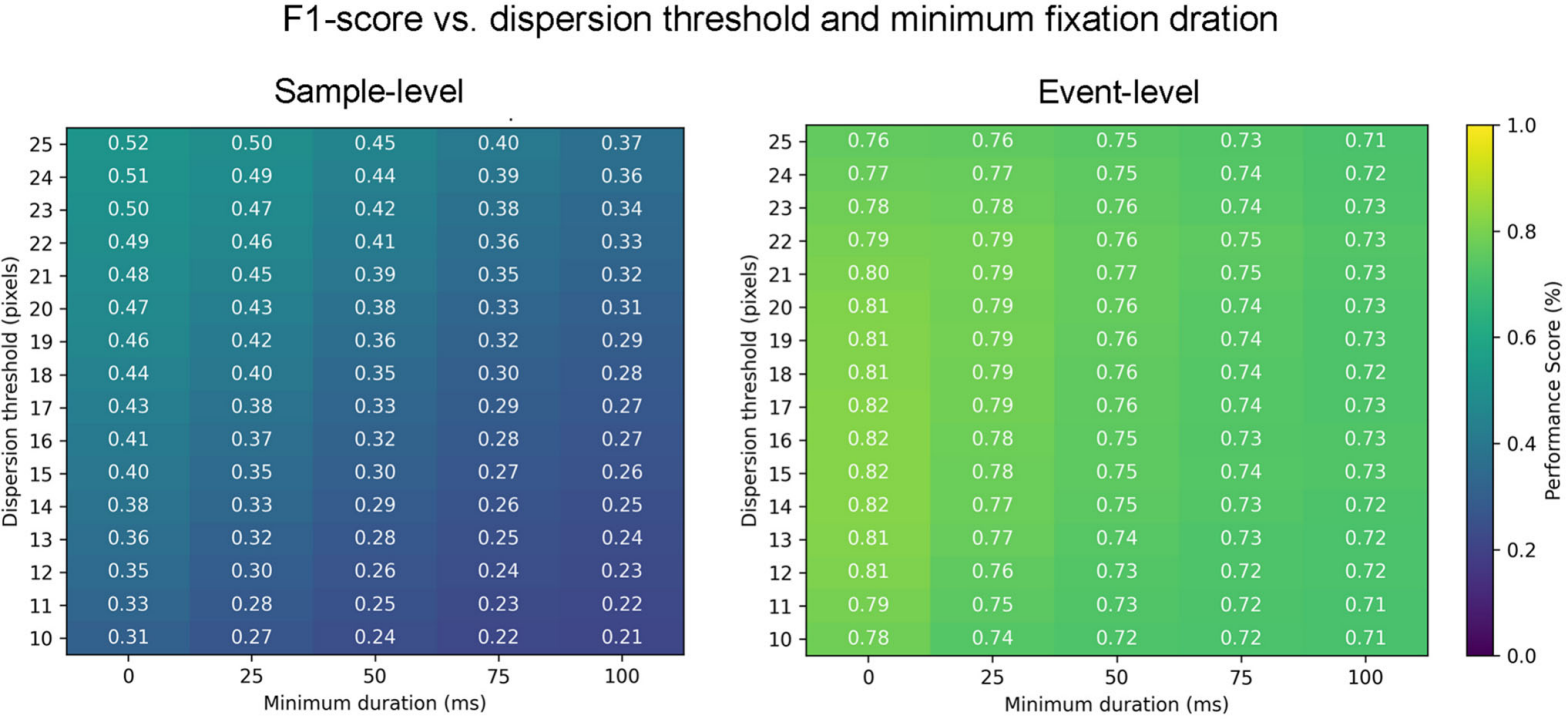
Performance of the I-DT algorithm across a range of parameter values, shown separately for mean sample-level and event-level F1-scores for predicting both sets of labels provided by the two annotators in our dataset. The heatmaps visualize how variations in the dispersion threshold and minimum fixation duration affect performance. *Warmer colors* (closer to yellow) indicate higher F1-scores, while *cooler colors* (closer to blue) indicate lower scores

### Threshold-based methods

#### Dispersion-threshold identification method (I-DT)

Among the threshold-based algorithms, the I-DT (Identification by Dispersion Threshold) algorithm is one of the simplest and most intuitive approaches. It classifies gaze points as either fixations or saccades by evaluating the spatial dispersion of consecutive gaze samples. When gaze points are confined within a defined spatial region (typically around 0.5$^{\circ }$ of visual angle) for at least a minimum duration, they are labeled as part of a fixation (Stark, 1981). Saccades, by contrast, are implicitly defined as all other data points.

The I-DT algorithm relies on two key parameters: the dispersion threshold and the duration threshold. The dispersion threshold usually ranges between 0.5$^{\circ }$ and 1$^{\circ }$ of visual angle. It can be estimated empirically through data exploration. In screen-based studies, the duration threshold typically ranges between 100 and 200 ms, depending on the cognitive demands of the task. However, given that the datasets in this study involve mobile eye-tracking, we performed our own optimization of this parameter to better suit the characteristics of such data.

Dispersion is calculated using a simple formula that sums the range of *X* and *Y* coordinates within a moving window:

2 $$\begin{aligned} D = [max(x) - min(x)] + [max(y) - min(y)]. \end{aligned}$$

Despite their simplicity, dispersion-based methods like I-DT can struggle in noisy environments. Determining optimal threshold values is critical, as poor parameter choices can significantly disrupt classification performance. A too high dispersion threshold will lead to false fixations and an underestimation of the number of gaze shifts, whereas a too low threshold might overlook genuine fixations and overestimate the number of gaze shifts. As such, parameter tuning in I-DT is critical, with substantial implications for detection performance and reliability (Blignaut, 2009).

We implemented the I-DT algorithm using the publicly available code provided by Birawo and Kasprowski (2022). To determine the optimal dispersion threshold, we conducted a parameter sweep on one of our recordings, evaluating performance against the manual annotations from both labelers. Figure 4 illustrates the algorithm’s performance across a range of dispersion threshold values and minimum fixation durations on our own dataset. Based on this analysis, a dispersion threshold of 22 pixels (about 1.7$^{\circ }$ visual angle) and no minimum fixation duration constraint were selected as the optimal setting for subsequent evaluations. Although it would have been possible to select a lower threshold for event-based evaluation and a higher one for sample-based evaluation, we opted to use a single threshold value for both. This decision was made to reflect a more realistic, practical application scenario in which you do not have labeled data to optimize the parameters. Using a similar process, we chose a dispersion threshold of 20 pixels and a minimum fixation duration of 5 ms for the Drews and Dierkes dataset.

#### Velocity-threshold identification method (I-VT)

Similar to the I-DT, the Identification by Velocity-Threshold (I-VT) algorithm (Salvucci & Goldberg, 2000) is another widely used method and serves as a foundation for automated and objective event detection in a lot of eye-tracking research. It is based on the principle that saccadic eye movements exhibit significantly higher velocities than fixations. Eye movement velocity profiles typically reveal a bimodal distribution, with low velocities corresponding to fixations and high velocities to saccades. The I-VT algorithm determines the velocity between consecutive gaze points and classifies each as either a fixation or a saccade based on whether it exceeds a predefined velocity threshold (Salvucci & Goldberg, 2000).

**Fig. 5:**
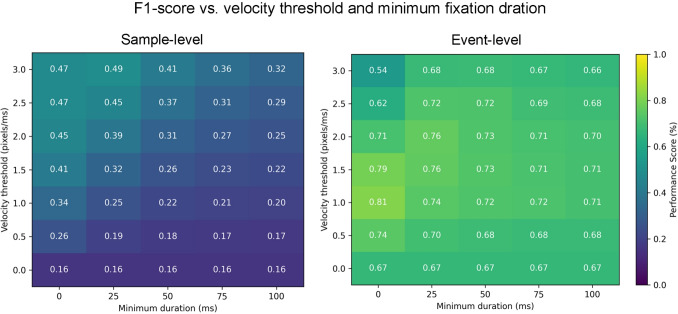
Performance of I-VT under different velocity thresholds and minimum fixation durations in our parameter sweep evaluation on our dataset. Same format as Fig. 4

In this paper, we used the implementation of I-VT from Birawo and Kasprowski (2022). To identify the optimal velocity threshold, we performed a parameter sweep on a representative recording, evaluating the algorithm’s performance against the manual annotations provided by both labelers. Figure 5 shows the performance of the I-VT algorithm on our own dataset across a range of threshold values and minimum fixation durations. Based on this analysis, a threshold of 2 pixels/ms (about 150$^{\circ }$/s and no enforcement of fixation duration were selected for use in all subsequent evaluations. With a similar process, we chose a velocity threshold of 2 pixels/ms and a minimum fixation duration of 15 ms for the Drews and Dierkes dataset.

#### Moving window method

The Moving Window algorithm is based on the method described by Hooge and Camps (2013) and is implemented in the open-source toolbox GazeCode (Benjamins, Hessels, & Hooge, 2018), which employs an adaptive velocity threshold technique. To estimate velocities, they fitted a parabola to every sequence of three consecutive gaze-position samples. The derivative of this parabola was used to compute the velocity at the central (second) sample in each trio. This process was applied throughout each gaze recording, excluding the first and last samples. A velocity-based algorithm identifies fixations and saccades by analyzing the velocity of gaze samples over time. Two key parameters govern its behavior: window size and lambda ($\lambda$). The window size determines the number of consecutive samples considered when evaluating potential fixations, with larger windows allowing for the detection of longer fixations and smaller windows being more sensitive to brief ones. The lambda parameter serves as a velocity threshold for saccade detection; eye movements exceeding this threshold are classified as saccades, whereas those below are candidates for fixations. By adjusting lambda, the algorithm’s sensitivity to rapid eye movements can be tuned: lower values increase sensitivity to small saccades but may increase false positives, whereas higher values yield more conservative saccade detection.

To isolate fixations, they calculated the mean and standard deviation of the absolute velocity, derived from both horizontal and vertical components, and removed all samples with velocities exceeding the mean plus three standard deviations. This thresholding process was iterated until the velocity threshold stabilized or until 50 iterations were completed. Afterwards, fixations shorter than 60 ms were excluded, and saccades smaller than 1.0$^{\circ }$ of visual angle were also removed. When such a small saccade was excluded, the fixations before and after it were merged.

**Fig. 6:**
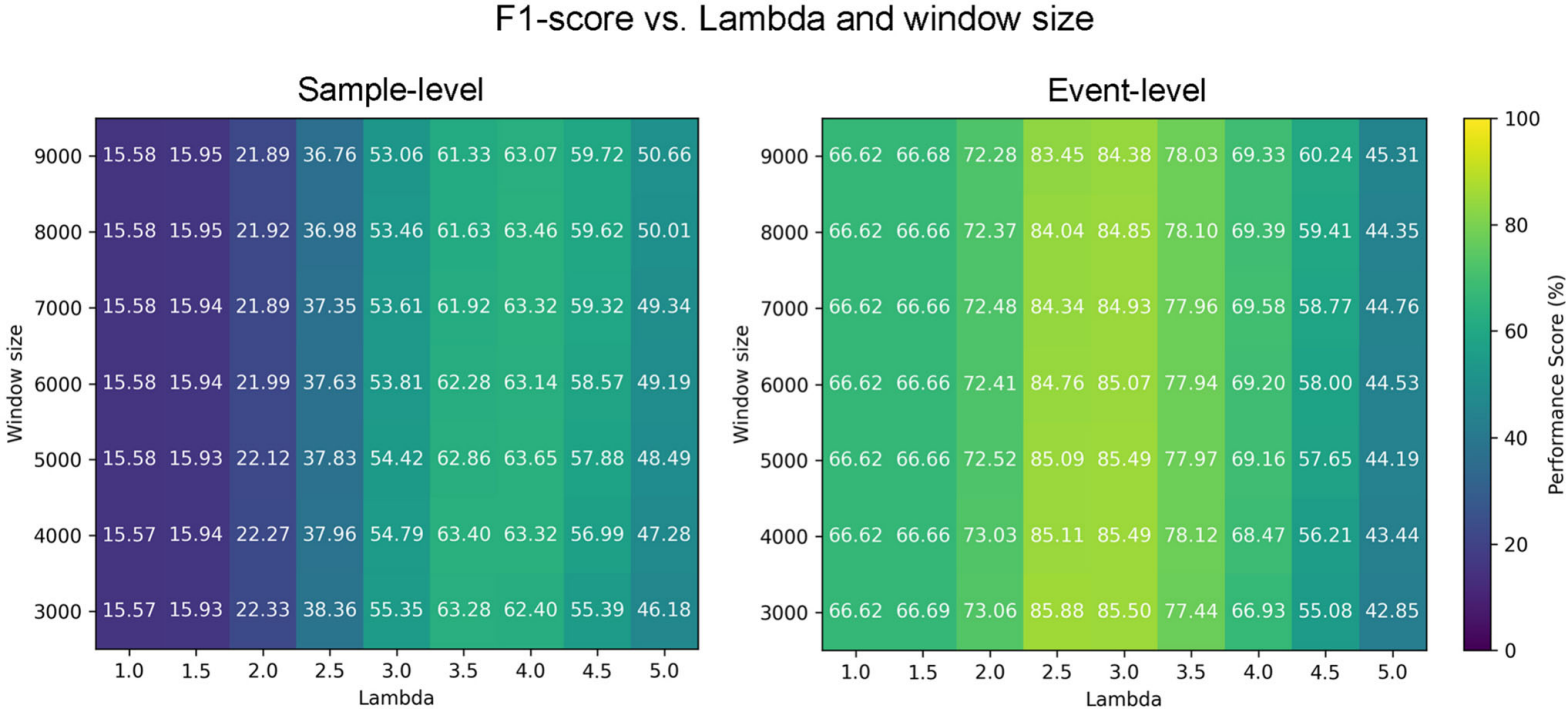
Performance of Moving Window algorithm under different lambda values and window sizes in our parameter sweep evaluation on our dataset. Same format as Fig. 4

In our evaluation, we used the implementation of this method provided by the authors (Benjamins et al., 2018). To identify the optimal parameters, including window size and lambda, we conducted a parameter sweep on a representative recording, evaluating the algorithm’s performance against the manual annotations provided by both of our labelers. Figure 6 presents the performance of the Moving Window algorithm across a range of parameter values, separately for sample- and event-level scores. Based on this analysis, we selected a lambda coefficient of 3.5 and a window size of 6000 samples (about half a minute) for all subsequent evaluations on our dataset. With a similar process, we chose a window size of 2000 samples and a lambda coefficient of three for the Drews and Dierkes dataset.

### Machine-learning-based methods

#### GazeNet

GazeNet (Zemblys et al., 2019) is an end-to-end deep learning framework developed for eye-movement event classification that bypasses the need for traditional hand-crafted features and manual threshold setting. Unlike earlier methods that rely on predefined velocity or dispersion criteria, GazeNet directly processes raw eye-tracking data to classify samples into fixations, saccades, and post-saccadic oscillations (PSOs). The model combines convolutional layers to extract local features and gated recurrent units (GRUs) to capture temporal dependencies, enabling the network to consider both short- and long-term context when predicting eye-movement events. This setup allows GazeNet to generate context-aware predictions that mimic expert-level human classification, without any post-processing or parameter tuning.

One major challenge GazeNet addresses is the limited availability of high-quality annotated training data. To overcome this, the authors developed a generative model called GazeGenNet, a recurrent neural network trained on a small manually labeled dataset. GazeGenNet synthesizes realistic eye-movement sequences that preserve both signal characteristics and event structure (e.g., PSOs only following saccades). These synthetic samples are then used to train GazeNet, significantly increasing the volume and variability of training data while maintaining biological plausibility. Performance evaluations showed that GazeNet not only achieved classification accuracy comparable to human coders but also generalized well across datasets collected from different eye trackers.

GazeNet was trained on the Lund2013 dataset (Andersson et al., 2017), which consists of monocular eye-movement recordings from participants viewing static images, dynamic videos, and moving dot stimuli shown on screen with a head-stabilizer. However, the authors speculate that their method is applicable on recordings from eye-trackers such as our dataset as well (Zemblys et al., 2019).

For the k-fold cross-validation, we retrained the model by initializing them with the original pre-trained weights and replacing the final classification layer with a new two-neuron layer to reflect the binary labeling scheme of our dataset, which differs from the multi-class configuration used in the original GazeNet study. This allowed us to assess model performance under fine-tuned conditions specific to our data.

#### Online eye-movement classification with temporal convolutional networks (OEMC)

The Temporal Convolutional Network (TCN) presented by Elmadjian et al. (2023) is a deep learning model tailored for real-time eye-movement classification, specifically the ternary classification of fixations, saccades, and smooth pursuits. Unlike traditional sequence models that rely on recurrent structures, the TCN uses stacked 1D convolutional layers with residual connections and exponentially increasing dilation rates to capture both short- and long-range temporal dependencies. Each temporal block in the TCN architecture consists of two convolutional layers and uses ReLU activation, with multiple dilation sizes to expand the receptive field without relying on future input, a key requirement for online classification.

To enhance TCN performance in online settings, the authors introduce a novel multi-scale, one-way feature-extraction method. This technique emphasizes the most recent data in a context window, avoiding future data leakage and enabling high-frequency, low-latency predictions. Their experiments show that the TCN achieves the highest F1 Scores across both sample and event levels when compared to similar methods.

OEMC was trained and evaluated on the GazeCom dataset (Startsev, Agtzidis, & Dorr, 2019) and the Head-Mounted Raw (HMR) dataset (Elmadjian et al., 2023). Although the HMR dataset was collected using a head-mounted eye tracker and GazeCom with a remote eye tracker, both datasets were recorded under controlled conditions while participants viewed stimuli on screens.

For the k-fold cross-validation, we used the pre-trained model published by the authors as the initialization for transfer learning purposes. The original multi-class output layer was replaced with a new two-neuron layer to adapt to the binary classification task in our dataset.

#### Automatic classification of gaze events in dynamic natural viewing (ACE-DNV)

ACE-DNV (Nejad et al., 2024), Automatic Classification of gaze Events in Dynamic Natural Viewing, is a machine learning framework designed to classify gaze events, such as fixation, smooth pursuit, gaze following while moving, and gaze shift, under natural and dynamic viewing conditions. It combines features derived from eye-movement signals, visual content changes at the point of gaze, and motion estimated through visual odometry to account for both eye and head/body movements.

To extract meaningful features, ACE-DNV computes eye-movement velocity and direction in a head-centered reference frame, measures scene content similarity using image patches around the gaze point using 2ch2stream neural network (Zagoruyko & Komodakis, 2015), and estimates head and body motion using a visual odometry method, known as DF-VO (Zhan, Weerasekera, Bian, & Reid, 2020). These features are interpolated and synchronized across the higher-frequency eye tracker and lower-frequency scene video. The resulting feature vectors, containing gaze velocity, head/body motion, and gaze patch similarity, are input into a random forest classifier, which labels each time point into one of the four gaze-event types.

ACE-DNV was trained and evaluated on the Gaze-in-Wild dataset, which includes recordings of participants freely moving through natural environments while performing tasks such as walking, throwing, and catching a ball, and conducting visual search. The method demonstrated competitive performance, achieving event-level F1-scores approaching the level of agreement between human annotators. Notably, it performs comparably to more complex systems like the Gaze-in-Wild BiRNN, while having fewer hardware dependencies. Its modular design allows researchers to extend the event categories or adapt it to new datasets, making it a flexible and accessible solution for naturalistic gaze analysis.

For the k-fold cross-validation of the Random Forest models, we initialized a new model from scratch in each iteration. Each model was trained on the binary labels from the five training recordings and evaluated on the isolated recording. Since the original models were trained on four-class labels, and due to the nature of Random Forests lacking transferable weight initialization, it was not possible to reuse the pre-trained models provided by the authors. Consequently, we retrained the models from scratch for each iteration.

### Ranking method

**Fig. 7:**
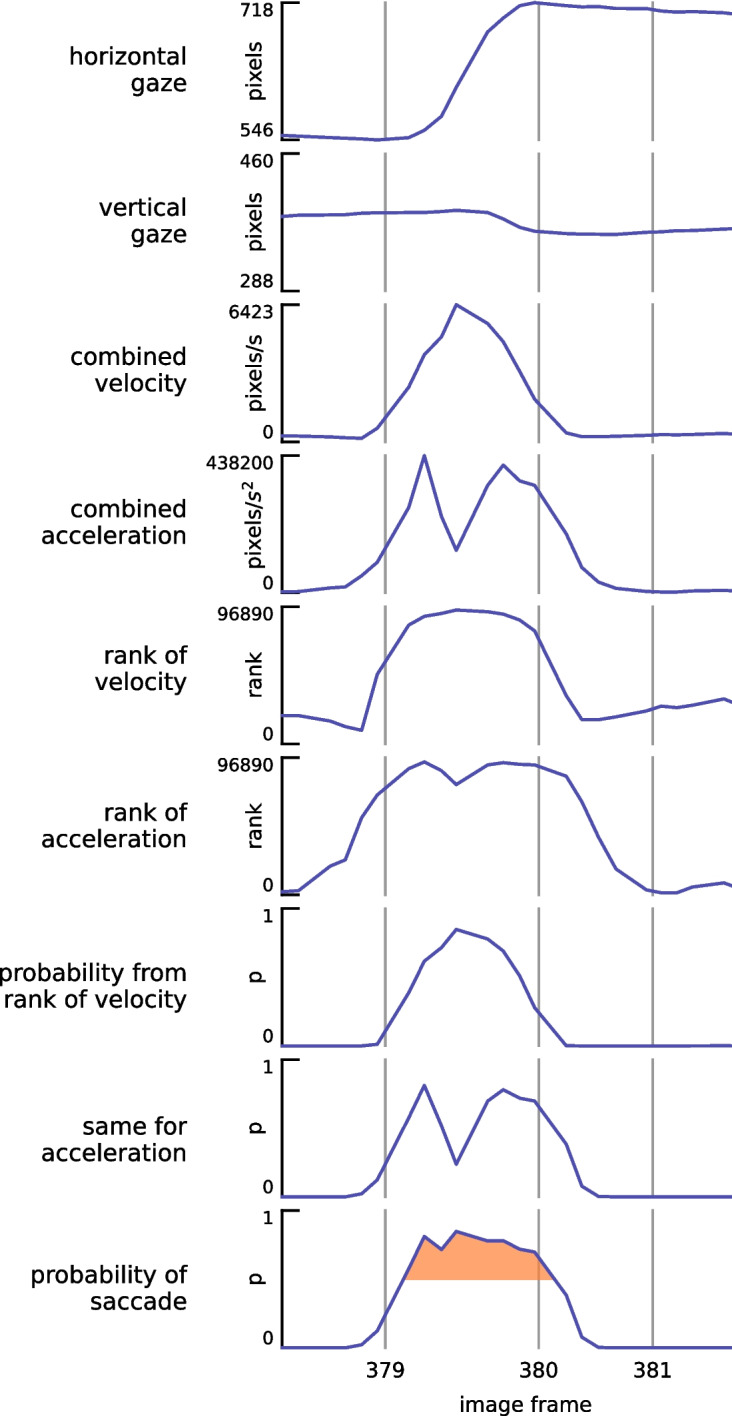
Example of the steps in saccade identification from the measured eye-in-head gaze data. The horizontal and vertical gaze orientations were first used to determine the velocity and acceleration of the eye’s rotation in the head, combining both horizontal and vertical gaze. The velocities and accelerations were then ranked, and the ranks were converted to probabilities through a power function such that only the 10% highest ranks have a value above 0.5. The highest of these two probabilities was taken as the probability of there being a saccade at each moment (highlighted on the bottom panel). The frame numbers correspond with those in Fig. 8

In this paper, we introduce the Ranking method for detecting gaze shifts, which combines two core principles. First, gaze shifts are characterized by rapid rotations of the eyes in the head. Second, gaze shifts typically serve to reorient gaze between distinct items of interest. Rather than applying a fixed velocity or acceleration threshold, we rank velocity and acceleration values across the entire dataset and relate these ranks to the likelihood of a gaze shift at each point in time (Fig. 7).

**Fig. 8:**
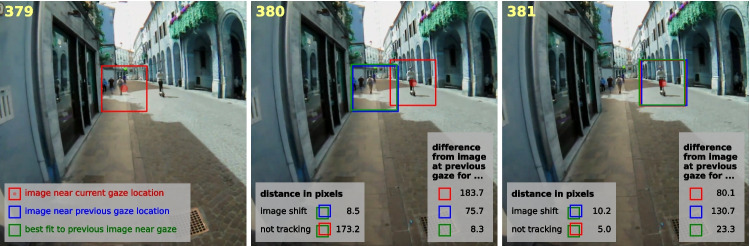
Example of scene-based saccade identification for three consecutive frames (379–381). *Red squares* mark image regions centered on the current gaze position, *blue squares* mark image regions centered on the former gaze position, and *green squares* mark the best-matching region corresponding to the previously fixated patch. Between frames 379 and 380, gaze shifts from the person on the sidewalk to the person on the street, leading to relatively high values for both the not-tracking distance (173.2 px) and the difference between the previous image at gaze and the current image at gaze (183.7). Between frames 380 and 381, gaze tracks the person on the street, so the not-tracking distance has a relatively low value (5.0 px), and the difference between the previous image at gaze and the current image at gaze (80.1) is smaller than the difference between the same positions in the image (130.7)

We compute velocity and acceleration at each moment of interest by fitting a second-order polynomial to the eye-movement data within a 40-ms window centered on that moment (excluding blinks; Savitzky–Golay filter). We then rank the velocity and acceleration values from smallest to largest, assigning the smallest rank a probability of 0 and the largest rank a probability of 1. The probability function is formulated as a power function of the rank divided by the total number of measurements, with the exponent selected so that only the 10% highest ranks have a probability that is larger than 0.5 (an exponent of about 6.58). This procedure is performed separately for velocity (v) and acceleration (a), and the final probability at each moment is taken as the larger of the two values. This approach captures the fact that acceleration is highest near the onset and offset of a gaze shift, whereas velocity peaks in the middle.

Hence, we define fast eye-movement probability, $p_{eye}(t)$ for each time point *t* as

3 $$\begin{aligned} \text {for } m \in \{v,a\}: p_m(t)=(\frac{rank_m(t)}{N})^\alpha , \end{aligned}$$

4 $$\begin{aligned} p_{eye}(t) = max\{p_a(t), p_v(t)\}, \end{aligned}$$

where $rank_m(t)$ is the rank of the velocity ($m=v$) or acceleration ($m=a$) at time *t* when all measurements are sorted (from 1 for the slowest to *N* for the fastest), *N* is the total number of measurements, and $\alpha$ is the exponent that results in (only) the top 10% of measurements having $p_m(t)>0.5$.

To evaluate whether gaze shifts between items in the scene, we compared the visual content around three places in each image frame with the visual content around the gaze point in the previous image frame. The visual content was specified by a square image patch, with sides of approximately 15$^{\circ }$, centered at the three places of interest (Fig. 8). These three places were the gaze position in the current frame, the gaze position in the previous frame, and the location in the current image that best matched the previously fixated region. The best-matching patch was found by sliding the patch centered on the gaze position in the previous frame across the current frame and identifying the most similar region based on squared RGB differences.

Such squared RGB differences were also used to evaluate the differences between the image patches. If gaze remains on the same item, the image at gaze should be similar across consecutive frames, so the patch at gaze at time *t-1* should be similar to the patch at gaze at time *t*. If gaze shifts to another item, the two patches should be different. However, the magnitude of the difference does not only depend on the change in gaze. For instance, if the overall illumination suddenly changes, the RGB difference will be large even if the gaze does not change. Conversely, if one shifts gaze between two tiny specks on a uniform background the difference will be small. We therefore compared the difference between the image at gaze on consecutive trials, with the difference that one would obtain if gaze had not shifted. Moreover, we did not consider the differences themselves, but how much larger those differences were than the smallest possible difference.

Let $\Delta (.,.)$ denote the squared RGB difference between two patches. Further, let *s*(*a*, *b*) denote the square patch of the image on frame *a* at the gaze position on frame *b*, and *t* denote the current frame. Then, $s(t-1,t-1)$ is the patch of the previous image near the gaze position on the previous frame, *s*(*t*, *t*) is the patch of the current image at gaze on the current frame (red squares in Fig. 8), and $s(t,t-1)$ is the patch of the current image at the gaze location on the previous frame (blue squares). Finally, let $b(t,t-1)$ be the patch of the current frame that best matches the patch at gaze on the previous frame (green squares), as found by sweeping the 15$^{\circ }$ square region at gaze on frame *t*-1 across the image on frame *t.* We determine how much worse the patch at gaze in the previous image matches the patches at the current ($d_{current}$) and previous ($d_{previous}$) gaze in the current image than it could have (i.e. than the best match), and use this to compute a probability of there having been a saccade between the previous and the current image:

5 $$\begin{aligned} d_{current}(t) = \Delta (s(t,t), s(t-1,t-1))-\Delta (b(t,t-1), s(t-1,t-1)) \end{aligned}$$

6 $$\begin{aligned} d_{previous}(t) = \Delta (s(t,t-1), s(t-1,t-1))-\Delta (b(t,t-1), s(t-1,t-1)) \end{aligned}$$

7 $$\begin{aligned} p_{scene,1}(t) = \frac{d_{current}(t)}{d_{current}(t)+d_{previous}(t)}. \end{aligned}$$

If $d_{current}(t)$ is small, meaning that the match at gaze is similar to the best match in terms of image similarity, the probability that a saccade has taken place is small. The match at gaze is compared with the match at the old gaze position $d_{previous}(t)$ to account for the fact that the difference between the RGB values depends on their variability in the image.

We also computed a second probability measure based on the above-mentioned matching strategy, but focused on how gaze moves across the scene, rather than how the scene changes around the gaze location. This metric is designed to distinguish between fast eye-in-head movements that maintain gaze on a single object of interest, and those that shift gaze to a different object. When the eyes track a moving object, either because the head moves or the object itself moves, the gaze position shifts across the image in a similar way as the best-matching region from the previous frame. In such cases, the distance between the current gaze position and the predicted region (i.e., the region that best matches the previous gaze location) remains small. However, when gaze shifts to a new object, this distance becomes large. To reduce sensitivity to potential errors in matching, we normalize this distance by the distance between the previous gaze position and the best-matching region in the current frame.

Let D(a,b) denote the distance between a and b (within the image) and *g*(*t*) denote the gaze location in frame t (in image coordinates), and *m*(*t*) denote the position on the current frame that best matches the position at gaze on the previous frame (the position of $b(t,t-1)$ ). We compared the distances of the current gaze position *g*(*t*) and of the former gaze position $g(t-1)$ from the best match *m*(*t*)) to compute a second measure of the probability of there having been a saccade between the previous and the current image:

8 $$\begin{aligned} p_{scene,2}(t) = \frac{D(g(t),m(t))}{D(g(t),m(t)) + D(g(t-1),m(t))}. \end{aligned}$$

If the scene hardly shifts in the image (for instance, because the head hardly moves), but a saccade changes the direction of gaze (see change from frame 379 to frame 380 in Fig. 8), *D*(*g*(*t*), *m*(*t*)) will be large (*not tracking*) and $D(g(t-1),m(t))$ will be small (*image shift*), so $p_{scene,2}(t)$ will be large. If gaze follows a moving patch in the image, *D*(*g*(*t*), *m*(*t*)) will be small, so $p_{scene,2}(t)$ will be small (frame 381 in Fig. 8).

We combine these two scene-based probabilities by multiplying them and taking the square root:

9 $$\begin{aligned} p_{scene}(t) = \sqrt{p_{scene,1}(t) \times p_{scene,2}(t)}. \end{aligned}$$

To consider the scene information, we combine the eye movement-based probability $p_{eye}(t)$ with the scene-based probability $p_{scene}(t)$, again by multiplying them and taking the square root:

10 $$\begin{aligned} p_{gaze}(t)=\sqrt{p_{scene}(t) \times p_{eye}(t)}. \end{aligned}$$

Any moment for which the probability exceeds 0.5 is marked as a potential component of a gaze shift. Once all potential components are identified, we group consecutive moments to define individual gaze shifts. A group is only considered a true gaze shift if the total eye rotation exceeds 1$^{\circ }$. Any segments whose angular direction deviates by more than 90$^{\circ }$ from the overall direction of the putative gaze shift are excluded. We also enforce a minimum interval of 50 ms between shifts. If two shifts appear within 50 ms and their directions differ by less than 45$^{\circ }$, we merge them into a single gaze shift; otherwise, the smaller one is discarded. The resulting list of intervals reflects all detected gaze shifts under these criteria. We evaluated our proposed method using both eye-based probabilities ($p_{eye}$) and combined eye- and scene-based probabilities ($p_{gaze}$), allowing us to assess the contribution of scene-based information to overall performance.

The Python implementation of the proposed Ranking method is available in the code repository accompanying this paper^1^.

## Results

This section presents the results obtained from our study. To assess inter-labeler agreement, we applied the same evaluation used to compare the various methods to a single labeler to the manual annotations of the two labelers in our own dataset. This showed sample- and event-level F1-scores of about **72%** and **86%**, respectively, which can be considered empirical benchmark scores.

Table 1 summarizes the performance of various gaze event identification methods, comparing their sample-level and event-level F1-scores on our dataset using annotations from two independent labelers (EB and AG) averaged across all recordings. The table also provides results for the Drews and Dierkes dataset using their labels, denoted as “D&D” dataset. Table 2 reports the false-positive and false-negative rates for each method at the sample- and event-level, separately for each annotator.

**Fig. 9:**
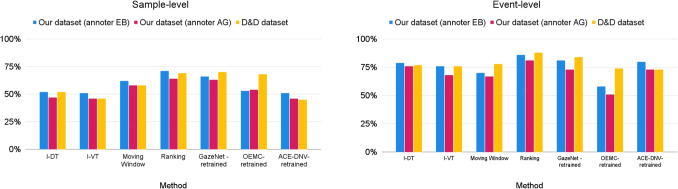
F1-scores of all methods evaluated in this study, comparing optimized threshold-based methods, retrained machine-learning-based methods, and the proposed Ranking method when incorporating both eye-in-head movements and visual scene analysis ($P_{gaze}$). The left and right panels show sample-level and event-level F1-scores, respectively. Results are shown separately for three subsets: our dataset labeled by EB (*blue*), our dataset labeled by AG (*red*), and the Drews and Dierkes dataset (*yellow*), denoted as “D&D” in the figure

**Fig. 10:**
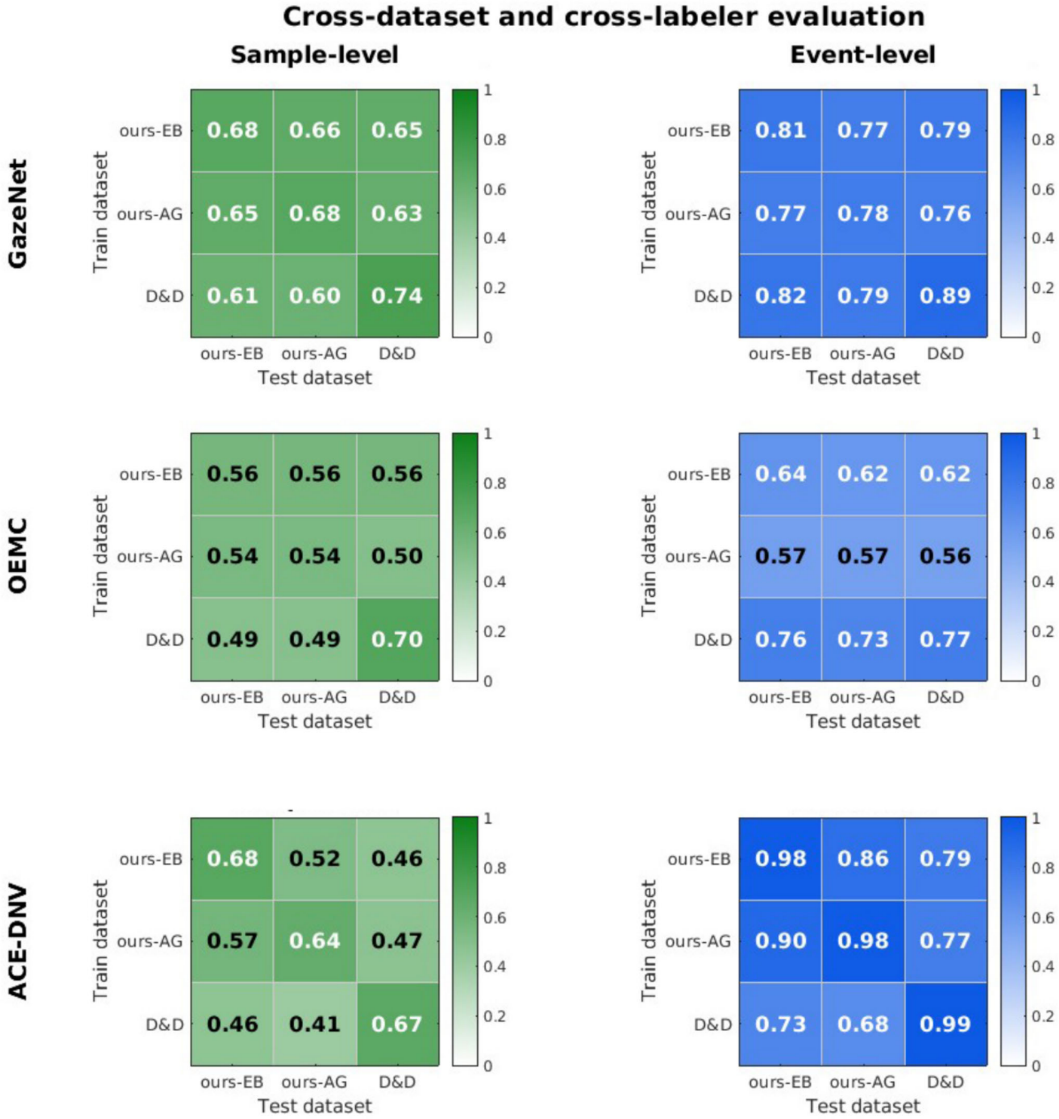
Cross-dataset and cross-labeler generalizability of machine-learning-based methods. Each matrix shows the F1-scores of a model trained on a dataset annotated by a single labeler (*rows*) and evaluated on different datasets and annotators (*columns*). The left column displays sample-level F1-scores (*green*), and the right column shows event-level F1-scores (*blue*). Results are reported for the three machine-learning-based methods: ACE-DNV (*top*), GazeNet (*middle*), and OEMC (*bottom*)

Table 3 presents the average sample- and event-level scores achieved by the retrained machine-learning-based methods using a k-fold cross-validation process, while Table 4 reports the corresponding average false-positive and false-negative rates. Figure 9 summarizes the provided tables to illustrate the performance of all methods with optimized thresholds and retrained machine learning models. Figure 10 reports the results achieved by each machine-learning-based method in the cross-dataset and cross-labeler generalizability evaluation.

**Table 1:** Sample-level and event-level F1-scores of the gaze shift identification methods that we considered. Shown separately for *our dataset* labeled by annotator EB, *our dataset* labeled by annotator AG, and the *Drews and Dierkes dataset*, using their original labels. The scores are averaged across all recordings in each dataset

Method	Our dataset		Our dataset		D&D dataset	
	(annotator EB)		(annotator AG)		(their labels)	
	sample	event	sample	event	sample	event
I-DT	52%	79%	47%	76%	53%	80%
I-VT	51%	76%	46%	68%	46%	75%
Moving Window	62%	70%	58%	67%	59%	76%
Ranking ($p_{eye}$)	71%	78%	64%	73%	48%	76%
Ranking ($p_{gaze}$)	71%	86%	63%	81%	69%	88%
GazeNet	18%	68%	15%	67%	21%	67%
OEMC	7%	1%	7%	1%	7%	1%
ACE-DNV	21%	61%	18%	59%	23%	63%

**Table 2:** false-positive (FP) and false-negative (FN) rates for each method at the sample- and event-level, reported separately for each dataset and labeler. Values indicate the percentage of misclassifications relative to the total number of samples or events within each category (e.g., FN is the proportion of gaze shift instances classified incorrectly as non-gaze-shifts)

Method	Our dataset				Our dataset				D&D dataset			
	(annotator EB)				(annotator AG)				(their labels)			
	sample		event		sample		event		sample		event	
	FP	FN	FP	FN	FP	FN	FP	FN	FP	FN	FP	FN
I-DT	12%	36%	9%	29%	13%	34%	8%	33%	11%	38%	4%	29%
I-VT	26%	23%	21%	17%	27%	22%	19%	25%	24%	26%	18%	28%
Moving Window	4%	39%	6%	41%	6%	38%	5%	46%	7%	36%	5%	34%
Ranking ($p_{eye}$)	3%	31%	2%	34%	5%	31%	2%	41%	3%	36%	1%	32%
Ranking ($p_{gaze}$)	6%	21%	3%	22%	8%	21%	3%	29%	5%	27%	1%	20%
GazeNet	95%	0%	98%	0%	95%	0%	98%	0%	94%	0%	99%	0%
OEMC	1%	96%	0%	99%	1%	96%	0%	99%	1%	96%	0%	99%
ACE-DNV	82%	18%	90%	16%	82%	18%	90%	18%	94%	0%	99%	0%

## Discussion

### Performance of gaze shift identification methods

The main finding of this study is that traditional threshold-based methods outperformed current pre-trained machine-learning-based approaches when applied to gaze data collected in dynamic, real-world conditions using head-mounted eye trackers. While machine learning methods have shown strong performance in controlled, screen-based settings, our results suggest that their generalizability to naturalistic environments remains limited. However, we also show that they could be trained to perform much better. Notably, our newly proposed Ranking method outperformed the other methods across both sample-level and event-level evaluations, indicating its potential as an alternative for gaze-shift detection in mobile gaze analysis.

### Threshold-based methods

Among the threshold-based methods, overall performance varied across sample- and event-level evaluations. Although the Moving Window method showed relatively better sample-level performance, the I-DT algorithm achieved the highest event-level score. These results suggest that different threshold-based methods may be more or less suited depending on the desired level of temporal resolution in gaze shift detection.

The relatively high event-level but low sample-level scores of I-DT are likely due to its sensitivity to short-term fluctuations and parameter dependencies. Sample-level misclassifications, which may occur at the onset and offset of saccades, have a limited impact on event-level performance, as this is determined by majority voting across samples. These results underscore that, while useful, threshold-based methods like I-DT can be prone to brief misclassifications at the sample level.

Among threshold-based methods, the Moving Window approach demonstrated the lowest average false-positive rates, 5% at both the sample- and event-level, indicating a conservative classification strategy. However, it also exhibited the highest false-negative rates within this category of methods, about 38% at the sample-level and about 43% at the event-level on our dataset, suggesting a tendency to misclassify gaze shift samples and events as non-gaze-shifts. This trade-off highlights the method’s inclination to prioritize precision over recall in detecting gaze shifts.

In this study, we had access to manual annotations and used one-sixth of the recording to optimize the parameters of each method before applying them to the full dataset. Therefore, the observed performance may be influenced by the parameter settings chosen in this study. However, in studies lacking ground truth labels, identifying suitable parameters may be more difficult, potentially limiting the reliability of threshold-based methods in such contexts. By contrast, adaptive methods, such as the Ranking method, may offer easier implementation without a need for prior extensive parameter tuning.

**Table 3:** Mean F1-scores for the machine-learning-based algorithms when retrained using a k-fold cross-validation, shown separately for each dataset and labeler

Method	Our dataset		Our dataset		D&D dataset	
	(annotator EB)		(annotator AG)		(their labels)	
	sample	event	sample	event	sample	event
GazeNet	66%	81%	63%	73%	69%	85%
OEMC	53%	58%	54%	51%	63%	61%
ACE-DNV	51%	80%	46%	73%	46%	76%

**Table 4:** Mean false-positive (FP) and false-negative (FN) rates for the retrained machine-learning-based algorithms, shown separately for each dataset and labeler

Method	Our dataset				Our dataset				D&D dataset			
Method	(annotator EB)				(annotator AG)				(their labels)			
	sample		event		sample		event		sample		event	
	FP	FN	FP	FN	FP	FN	FP	FN	FP	FN	FP	FN
GazeNet	3%	37%	1%	31%	2%	42%	0%	41%	3%	35%	0%	25%
OEMC	3%	55%	1%	58%	2%	65%	0%	73%	4%	48%	2%	55%
ACE-DNV	16%	28%	10%	26%	16%	29%	7%	37%	18%	36%	18%	36%

In the optimization process for the threshold-based methods, we selected parameters using one-sixth of the recordings and then applied the methods to the entire dataset to evaluate performance. This approach was chosen to simulate a practical scenario in which a user labels only a small subset of their data for parameter tuning, aiming to assess how well the method generalizes to the full dataset. However, we acknowledge that this setup introduces a degree of information leakage, as the optimization and evaluation phases are not fully independent.

### Machine-learning-based methods

#### Pre-trained models evaluation

When using pre-trained models, machine learning-based methods (GazeNet, OEMC, and ACE-DNV) showed relatively poor overall performance on our dataset. Among the machine-learning-based methods, OEMC yielded particularly low scores, indicating limited suitability for our data. Looking at the false-positive and false-negative rates revealed that all three machine learning models tended to converge toward predicting a single dominant class. Specifically, ACE-DNV and GazeNet predominantly classified samples as gaze shifts, whereas OEMC consistently predicted non-gaze-shift labels. The poor performance of the machine-learning-based methods is likely due to differences between our datasets and those used to train these models, highlighting a key limitation: their lack of generalizability. They may be too sensitive to dataset differences such as variations in the conducted tasks, experimental setups, or even environmental factors such as lighting conditions.

#### K-fold evaluation

Retraining the machine-learning-based methods demonstrated their potential when trained and tested on the same dataset. Our k-fold cross-validation showed that all these models can effectively learn and replicate previously observed labels, with GazeNet achieving the highest sample-level F1-scores of 66% and 63% on our dataset, and 69% on Drews and Dierkes dataset. For event-level scores, GazeNet and ACE-DNV achieved high event-level F1-scores (about 81% and 73%) on our dataset. Performance of GazeNet achieved the highest event-level F1-scores when trained and tested on Drews and Dierkes dataset. For our dataset, ACE-DNV produced fewer false negatives but more false positives in the gaze shift category compared to GazeNet. For Drews and Dierkes’ dataset, GazeNet had fewer false-negatives than ACE-DNV. The difference between the datasets could be due to differences in the participants’ tasks, or to the labels that were used during labeling, or to the different blink elimination procedures used for the two datasets.

#### Cross-dataset/labeler evaluation

To further assess generalizability, we trained each machine-learning model on the full dataset from a single annotator and tested it across all datasets and labelers. This process, the results of which are shown in Fig. 10, reflects realistic deployment scenarios where annotated data may not be available for retraining. Comparing these results with the within-dataset k-fold validation (Table 3), we observe a difference in robustness across models.

GazeNet demonstrated stable performance in cross-dataset and cross-labeler conditions, with sample-level F1-scores ranging from 60% to 66%, slightly lower than the k-fold results (63–69%). Event-level F1 scores in cross-dataset and cross-labeler conditions ranged from 76% to 82%, which is very similar to the k-fold results (73% to 85%). Thus, GazeNet is relatively robust to differences between datasets and annotator styles, making it a promising option for applications where retraining may not be feasible.

OEMC seems to have learnt something very general, because it did not always perform best when trained and tested on the same dataset. In general, performance appeared to depend more on the training dataset than the testing dataset. The sample-level performance (49–56%) was poorer than the sample-level k-fold scores of 53–63%. But the event-level performance (56–76%) was slightly better than the event-level k-fold scores of 51%-61%. Thus, OEMC appears to pick up on generalizable patterns but to be quite sensitive to the choice of training data.

ACE-DNV, exhibited greater variability across conditions. Sample-level performance transferred as well across the two annotators from our dataset (52% and 57%) as it did to different trials by the same labeler (k-fold scores of 46% and 51%). However, performance transferred less well across datasets, with performance dropping to 41–47%. At the event level, ACE-DNV showed better scores during cross-labeler testing (86% and 90%) than in k-fold validation (73–80%), possibly because the same recordings were all used for the training. The event-level F1-scores for cross-dataset tests ranged from 68% to 79%, slightly lower than the k-fold scores. Thus, ACE-DNV is also quite robust to differences between datasets and annotator styles, but it appears to pick up on more dataset-specific features.

### Ranking method

Our Ranking method outperformed all other approaches, including the retrained machine-learning-based models, on both datasets. It achieved sample-level F1-scores of 71% and 63% on our dataset and 69% on Drews and Dierkes’ dataset, as well as event-level F1-scores of 86% and 81% on our dataset and 88% on Drews and Dierkes’ dataset. These results demonstrate that the Ranking method consistently achieved the highest performance in detecting gaze shifts across both evaluation criteria. These scores are comparable to the agreement scores among the manual labelers in our dataset. The evaluation indicated that the Ranking method generally provided the highest scores for detecting gaze shifts compared to established threshold-based and machine-learning-based methods.

A comparison of the Ranking method with and without the inclusion of scene information reveals that scene-based features contribute significantly to event-level performance, but not to sample-level performance, given the minimal impact observed for the latter on our dataset. However, on Drews and Dierkes’ dataset, the inclusion of scene information appeared to have a stronger impact even at the sample level, suggesting that visual context contributed more substantially to accurate classification in that dataset. Incorporating scene information, as defined in Eq. 10, substantially reduced false-negative rates for both datasets. This suggests that the use of visual information from the central field of vision enhances the model’s ability to correctly identify instances where gaze shifts have occurred.

The consistent performance across both datasets and performance evaluation levels suggests that the Ranking method may offer a practical and generalizable alternative for analyzing gaze data collected from head-mounted eye trackers.

### Observations on gaze behavior

Some observations that we made during the manual labelling highlight the challenges in classifying eye movements during unconstrained, natural viewing tasks. While manually annotating gaze shifts, we noted various phenomena that are not often reported in laboratory-based eye-tracking research. Normally, saccades are understood as gaze shifts between distinct items or points of interest in the environment, while fixations are periods when gaze remains relatively stable on an object (Hessels, Niehorster, Nyström, Andersson, & Hooge, 2018). In dynamic contexts, fixations on moving targets are usually classified as smooth pursuit, whereas fixations by moving observers on static objects are often described in other terms, including the slow phases of the vestibulo-ocular reflex (VOR) or optokinetic nystagmus (OKN), depending on whether gaze stabilization results primarily from vestibular or visual information. However, during the current annotation process, we regularly observed instances where gaze did not consistently adjust to the retinal motion at the fixated scene location. Specifically, when participants walked along streets or across open squares, they often maintained their gaze fixed in a particular direction (generally straight ahead), allowing the visual scene to shift across the retina without continuous corrections. This behavior, of gaze shifts in the scene without the eyes rotating in the head, complicates the distinction between fixations and gaze shifts.

Another observation involves gaze shifts accompanying large head movements. While head rotations typically trigger alternating slow and fast gaze shifts, traditionally attributed to VOR or OKN, differentiating these two phases was often difficult in our natural walking conditions. Although we consistently coded the fast components as saccades (assuming the slow component tracks stable positions relative to the rotating head and the fast component shifts gaze forward), the actual distinction was not always straightforward. We occasionally observed rapid gaze shifts in the opposite direction to the direction of the head rotation, a phenomenon less commonly described and more difficult to interpret using traditional categories.

We also observed frequent instances of more gradual gaze shifts, rather than the rapid eye movements traditionally labeled as saccades. In these situations, gaze seemed to drift slowly toward items of interest rather than repositioning gaze quickly. Such smooth, drift-like gaze behaviors blur the classical distinctions between fixation, smooth pursuit, and saccadic gaze shifts. Apparent drift has been reported to arise from changes in pupil size in many eye-tracking systems (Hooge, Niehorster, Hessels, Cleveland, & Nyström, 2021; Salari, Niehorster, Nyström, & Bednarik, 2025). This has not been studied for the Pupil Invisible, but the drifts that we observed were much larger than the drifts typically attributed to pupil-size effects. Additionally, gaze could smoothly transition from fixating a stationary object to pursuing a moving target entering the same spatial region, thereby changing the object of fixation without any clear saccadic movement.

Finally, we frequently observed changes in gaze position during blinks. This co-occurrence with saccades is reasonable, as both blinks and saccades temporarily disrupt visual input. We found that participants’ gaze often appeared at a different location immediately after blinking than it was before blink onset. Furthermore, based on the blink detection provided by the Pupil Invisible glasses, participants were recorded as blinking for 7% to 26% of the recorded time (with an average around 20%), a surprisingly high proportion. Closer inspection of the data suggests potential overestimation of blink occurrences. Specifically, many reported blinks included seemingly valid gaze estimates, particularly when participants looked upwards at statues on sunny days, possibly indicating squinting rather than actual blinking. However, without additional physiological measurements, this interpretation remains speculative. It is also possible that participants did actually blink a lot due to the bright sunlight in some cases. In our analyses, we excluded blinks detected by the Pupil Invisible system, as most of the evaluated methods were not specifically designed to identify blinks. This ensured a more consistent basis for comparison across methods.

### Future work

This study set out to assess whether machine learning techniques have effectively solved the problem of eye-movement classification, specifically for saccades, broadly defined in accordance with Hessels et al. (2018). Our findings indicate that existing machine learning models perform relatively poorly when directly applied to data collected in natural, outdoor environments, while participants walked through a town and looked at statues. This shows that machine learning algorithms need to be trained on data collected while people perform a wider variety of tasks under more diverse circumstances. To test this, machine learning models must be retrained on newly collected data under many circumstances. High performance can be achieved by retraining on the dataset of interest, but if achieving similar performance on other datasets requires additional training, and therefore additional manual annotation, the practical applicability of such models will be limited.

The reliance of supervised learning models on high-quality labeled data presents a significant challenge, as acquiring such data is costly and time-consuming due to the need for manual annotation by human experts. An interesting and urgent question for future research is therefore whether training machine learning models on diverse datasets can produce models that generalize more effectively across different tasks and circumstances.

A limitation of our work concerns the effect of the blink detections used in this study. The Pupil Invisible system reports a blink detection recall of approximately 0.95, but its performance may vary depending on factors such as lighting conditions and pupil visibility. In addition, the lack of eye videos in Drews and Dierkes’ dataset led us to use a different blink removal method for that dataset. The effect of the blink detectors on the results should be further investigated in future research.

## Conclusion

In this study, we systematically compared several widely used gaze-shift identification algorithms’ performance on datasets collected under natural viewing conditions using head-mounted eye trackers. Our findings indicate that machine learning methods currently perform quite poorly when applied to dynamic, real-world scenarios, likely due to their dependency on training data that do not sufficiently generalize across different contexts. Traditional threshold-based algorithms that are simpler and more interpretable showed reasonable performance but required careful parameter tuning, limiting their robustness across varied situations. Our newly proposed Ranking method shows potential benefits in combining scene-based and eye-movement information without heavily relying on strict thresholds or extensive training data. Moreover, observations made during the manual annotation revealed various gaze behaviors rarely reported in laboratory studies, emphasizing the complexity and diversity of natural viewing conditions, and the challenge faced by any evaluation of this behavior.

## Data Availability

All data and code supporting the findings of this study are available at https://github.com/arnejad/Gaze-Shift-Compare

## Footnotes

1 https://github.com/arnejad/Gaze-Shift-Compare

## References

[CR1] Andersson, R., Larsson, L., Holmqvist, K., Stridh, M., & Nyström, M. (2017). One algorithm to rule them all? an evaluation and discussion of ten eye movement event-detection algorithms. *Behavior Research Methods,**49*, 616–637. 27193160 10.3758/s13428-016-0738-9

[CR2] Bahill, A. T., Clark, M. R., & Stark, L. (1975). The main sequence, a tool for studying human eye movements. *Mathematical Biosciences,**24*(3–4), 191–204.

[CR3] Benjamins, J.S., Hessels, R.S., Hooge, I.T. (2018). Gazecode: Open-source software for manual mapping of mobile eye-tracking data. In *Proceedings of the 2018 ACM Symposium on Eye Tracking Research & Applications,* pp. 1–4.

[CR4] Birawo, B., & Kasprowski, P. (2022). Review and evaluation of eye movement event detection algorithms. *Sensors,**22*(22), 8810. 36433407 10.3390/s22228810 PMC9699548

[CR5] Blignaut, P. (2009). Fixation identification: The optimum threshold for a dispersion algorithm. *Attention, Perception, & Psychophysics,**71*, 881–895. 10.3758/APP.71.4.881 19429966

[CR6] Brenner, E., Janssen, M., de Wit, N., Smeets, J. B., Mann, D. L., & Ghiani, A. (2024). Running together influences where you look. *Perception,**53*(5–6), 397–400. 38409958 10.1177/03010066241235112 PMC11088211

[CR7] Cesari, M., Heidbreder, A., St. Louis, E. K., Sixel-Döring, F., Bliwise, D. L., Baldelli, L., ... Stefani, A. (2022) Video-polysomnography procedures for diagnosis of rapid eye movement sleep behavior disorder (rbd) and the identification of its prodromal stages: guidelines from the international rbd study group. Sleep *45*(3):zsab257. 10.1093/sleep/zsab257 34694408

[CR8] Drews, M., & Dierkes, K. (2024). Strategies for enhancing automatic fixation detection in head-mounted eye tracking. *Behavior Research Methods,**56*(6), 6276–6298. 38594440 10.3758/s13428-024-02360-0 PMC11541274

[CR9] Elmadjian, C., Gonzales, C., Costa, R. L. D., & Morimoto, C. H. (2023). Online eye-movement classification with temporal convolutional networks. *Behavior Research Methods,**55*(7), 3602–3620. 36220951 10.3758/s13428-022-01978-2

[CR10] Gestefeld, B., Koopman, J., Vrijling, A., Cornelissen, F. W., & de Haan, G. (2020). Eye tracking and virtual reality in the rehabilitation of mobility of hemianopia patients: a user experience study. *Vision Rehabilitation International,**11*(1), 7–19.

[CR11] Ghiani, A., Mann, D., & Brenner, E. (2024). Methods matter: Exploring how expectations influence common actions. *Iscience,**27*(3). 10.1016/j.isci.2024.109076 PMC10867666 38361615

[CR12] Hessels, R. S., Niehorster, D. C., Nyström, M., Andersson, R., & Hooge, I. T. (2018) Is the eye-movement field confused about fixations and saccades? a survey among 124 researchers. *Royal Society Open Science,**5*(8):180,502. 10.1098/rsos.180502 PMC6124022 30225041

[CR13] Hooge, I., & Camps, G. (2013). Scan path entropy and arrow plots: Capturing scanning behavior of multiple observers. *Frontiers in Psychology,**4*, 996. 24399993 10.3389/fpsyg.2013.00996 PMC3872074

[CR14] Hooge, I. T., Niehorster, D. C., Hessels, R. S., Cleveland, D., & Nyström, M. (2021). The pupil-size artefact (psa) across time, viewing direction, and different eye trackers. *Behavior Research Methods,**53*(5), 1986–2006. 33709298 10.3758/s13428-020-01512-2 PMC8516786

[CR15] Hooge, I. T., Niehorster, D. C., Nyström, M., Andersson, R., & Hessels, R. S. (2022). Fixation classification: How to merge and select fixation candidates. *Behavior Research Methods,**54*(6), 2765–2776. 35023066 10.3758/s13428-021-01723-1 PMC9729319

[CR16] Hoppe, S., & Bulling, A. (2016). End-to-end eye movement detection using convolutional neural networks. ArXiv Preprint arXiv:1609.02452.

[CR17] Komogortsev, O. V., & Karpov, A. (2013). Automated classification and scoring of smooth pursuit eye movements in the presence of fixations and saccades. *Behavior Research Methods,**45*, 203–215. 22806708 10.3758/s13428-012-0234-9

[CR18] Kothari, R., Yang, Z., Kanan, C., Bailey, R., Pelz, J. B., & Diaz, G. J. (2020). Gaze-in-wild: A dataset for studying eye and head coordination in everyday activities. *Scientific Reports,**10*(1), 2539. 32054884 10.1038/s41598-020-59251-5 PMC7018838

[CR19] Land, M. F. (1992). Predictable eye-head coordination during driving. *Nature,**359*(6393), 318–320. 1406934 10.1038/359318a0

[CR20] Land, M. F., & Hayhoe, M. (2001). In what ways do eye movements contribute to everyday activities? *Vision Research,**41*(25–26), 3559–3565. 11718795 10.1016/s0042-6989(01)00102-x

[CR21] Nejad, A., de Haan, G. A., Heutink, J., & Cornelissen, F. W. (2024). Ace-dnv: Automatic classification of gaze events in dynamic natural viewing. *Behavior Research Methods,**56*(4), 3300–3314. 38448726 10.3758/s13428-024-02358-8 PMC11133063

[CR22] Ohno, T., Mukawa, N., & Yoshikawa, A. (2002). Freegaze: a gaze tracking system for everyday gaze interaction. In *Proceedings of the 2002 Symposium on Eye Tracking Research & Applications,* pp. 125–132.

[CR23] Salari, M., Niehorster, D. C., Nyström, M., & Bednarik, R. (2025). The effect of pupil size on data quality in head-mounted eye trackers. *Behavior Research Methods,**58*(1), 17. 41339990 10.3758/s13428-025-02880-3 PMC12675653

[CR24] Salvucci, D.D., & Goldberg, J.H. (2000). Identifying fixations and saccades in eye-tracking protocols. In *Proceedings of the 2000 Symposium on Eye Tracking Research & Applications,* pp. 71–78.

[CR25] Sharma, P. K., & Chakraborty, P. (2024). A review of driver gaze estimation and application in gaze behavior understanding. *Engineering Applications of Artificial Intelligence,**133*(108), 117.

[CR26] Sharma, P. K., & Chakraborty, P. (2025). Evaluation of data collection and annotation approaches of driver gaze dataset. *Behavior Research Methods,**57*(6), 172. 40369353 10.3758/s13428-025-02679-2

[CR27] Stark, L. (1981). *Scanpaths revisited: Cognitive models, direct active looking* (pp. 193–226). Eye Movements: Cognition and Visual Perception.

[CR28] Startsev, M., & Zemblys, R. (2023). Evaluating eye movement event detection: A review of the state of the art. *Behavior Research Methods,**55*(4), 1653–1714. 35715615 10.3758/s13428-021-01763-7

[CR29] Startsev, M., Agtzidis, I., & Dorr, M. (2019). Characterizing and automatically detecting smooth pursuit in a large-scale ground-truth data set of dynamic natural scenes. *Journal of Vision,**19*(14), 10–10. 31830239 10.1167/19.14.10

[CR30] Stern, J. A., Walrath, L. C., & Goldstein, R. (1984). The endogenous eyeblink. *Psychophysiology,**21*(1), 22–33. 10.1111/j.1469-8986.1984.tb02312.x 6701241

[CR31] Stuart, S., Galna, B., Lord, S., Rochester, L., & Godfrey, A. (2014) Quantifying saccades while walking: validity of a novel velocity-based algorithm for mobile eye tracking. In *2014 36th Annual International Conference of the IEEE Engineering in Medicine and Biology Society,* IEEE, pp. 5739–5742. 10.1109/EMBC.2014.6944931 25571299

[CR32] Zagoruyko, S., & Komodakis, N. (2015). Learning to compare image patches via convolutional neural networks. In *Proceedings of the IEEE Conference on Computer Vision and Pattern Recognition,* pp. 4353–4361.

[CR33] Zemblys, R., Niehorster, D. C., Komogortsev, O., & Holmqvist, K. (2018). Using machine learning to detect events in eye-tracking data. *Behavior Research Methods,**50*, 160–181. 28233250 10.3758/s13428-017-0860-3

[CR34] Zemblys, R., Niehorster, D. C., & Holmqvist, K. (2019). gazenet: End-to-end eye-movement event detection with deep neural networks. *Behavior Research Methods,**51*, 840–864. 30334148 10.3758/s13428-018-1133-5

[CR35] Zhan, H., Weerasekera, C. S., Bian, J. W., & Reid, I. (2020) Visual odometry revisited: What should be learnt? In *2020 IEEE International Conference on Robotics and Automation (ICRA),* IEEE, pp. 4203–4210.

